# 3D‑printed training model for periodontal splinting: a randomized preclinical study

**DOI:** 10.1038/s41598-026-52988-5

**Published:** 2026-05-22

**Authors:** Christian Hoehne, Soeren Rehling, Johannes Schrenker, Yvonne Jockel-Schneider, Gabriel Krastl, Marc Schmitter

**Affiliations:** 1 Department of Prosthodontics, University of Wuerzburg, Pleicherwall 2, Wuerzburg, 97070 Germany; 2 Department of Conservative Dentistry and Periodontology, University of Wuerzburg, Pleicherwall 2, Wuerzburg, 97070 Germany

**Keywords:** 3D printing, Additive manufacturing, Dental education, Periodontal splinting, Fibre‑reinforced composite, Health care, Medical research

## Abstract

Preclinical simulation is essential for students to practice before clinical treatment. A low-cost 3D-printed model was developed with repositionable mobile maxillary anterior teeth to train periodontal splinting and evaluate learner perception and repositioning accuracy. In a two-period randomized cross-over course 43 students in their fourth year completed two palatal slot splints from the right to the left maxillary canine (FDI 13–23) in randomized material order. They used a polyethylene ribbon and a pre-impregnated unidirectional glass-fibre bundle. Outcomes included an electronic questionnaire using an anchored 6‑point ordinal scale from 1 for “Excellent” to 6 for “Unsatisfactory” following the German school grading system and geometric deviation of each tooth to an ideal position. The course was rated overall as good (Md 2.0, IQR 1.0) and highly clinically relevant (Md 1.0, IQR 1.0). Self-reported preparedness for splinting improved from poor (Md 5.0, IQR 1.0) before the course to good (Md 2.0, IQR 1.0; *p* < 0.001) after the course. In this pilot study, no statistically detectable positional deviations between materials or between the first and second session in this cohort were observed. This inexpensive, reproducible 3D-printed model enables realistic periodontal splinting training under simulated mobility conditions and may support structured preparation for clinical teaching.

## Introduction

Simulation using physical models enhances didactic instruction. It offers safe and repeatable practice of procedural steps before real patient treatment. Additive manufacturing has expanded dental simulation beyond typodonts. It enables the production of accurate models with realistic anatomy, surface quality and mechanical behaviour at low cost. Training models can be fabricated to specific learning situations. Across all dental disciplines - prosthodontics, endodontics, orthodontics, and oral surgery - in the last years 3D-printed teaching models have been developed to demonstrate and teach complex workflows^1–7^. However, periodontal practical training remains underrepresented despite its clinical relevance, especially when stabilizing mobile teeth with periodontal splinting.

The main reason for a splint is to distribute forces across multiple teeth to reduce pathological mobility^8,9^. The main utilized contemporary chairside approaches use fibre-reinforced *composites* (FRCs) made from polyethylene ribbons or pre-impregnated glass-fibre bundles^10–12^. After some minimal preparation they are bonded into a shallow palatal channel and covered with composite. Improved patient comfort and mobility reduction are reported, but the technique is sensitive.

In the present study a training model was developed which specifically targets the clinically important step of repositioning mobile anterior maxillary teeth and stabilizing them with a chairside fibre‑reinforced composite splint. In contrast to commercially and individual printed fixed typodont teeth, our printed anterior teeth are by design mobile and can be manually repositioned to an ideal arch before splint placement. They create a reproducible mobility and repositioning challenge. The teeth are designed to fit into a standard commercial study model, enabling adoption beyond a single institution. They can be produced at very low per‑tooth cost for course‑scale repetition. We also provide here an objective 3D deviation workflow that can be used for structured feedback on tooth repositioning accuracy. This model was evaluated under preclinical conditions with students. They performed two splinting exercises in randomized material order.

Feasibility, learner perception, and objective positional performance improvement were evaluated. The hypothesis was that students (1) perceive the model as realistic and clinically relevant; (2) handling might differ between the two fibre‑reinforced systems; and (3) a second session could improve repositioning accuracy. It must be acknowledged that the study is exploratory and may be underpowered for small effects.

## Methods

### Ethics approval

The study and experimental protocol were approved by the Institutional Review Board of the University of Wuerzburg (2026‑2‑ka). Participation was voluntary. Students were informed about the study procedures and provided informed consent prior to participation.

### Study design and setting

This study was conducted as a two‑period randomized cross‑over educational evaluation. The course was held within a preclinical periodontal splinting course. The cohort size was determined by usual class sizes. A total of 43 dental students (24 women, 19 men; mean age 25 years) participated voluntarily and had prior phantom‑head experience. They were all in their first clinical prosthodontics course.

Each student completed two splinting exercises on separate occasions which were two weeks apart. To counterbalance order effects students were randomly assigned to one of two sequence groups: They started with either polyethylene ribbon (Ribbond Ultra, Ribbond Inc., Seattle, Washington) (*n* = 21) or pre-impregnated unidirectional glass-fibre bundles (everStick PERIO, GC Germany GmbH, Bad Homburg, Deutschland) (*n* = 22). Sequence allocation was generated from a computer‑created randomization list prepared before the first session. Students worked independently and did not receive performance feedback prior to questionnaire completion and model scanning.

### CAD design and 3D-printing of training teeth

**Fig. 1:**
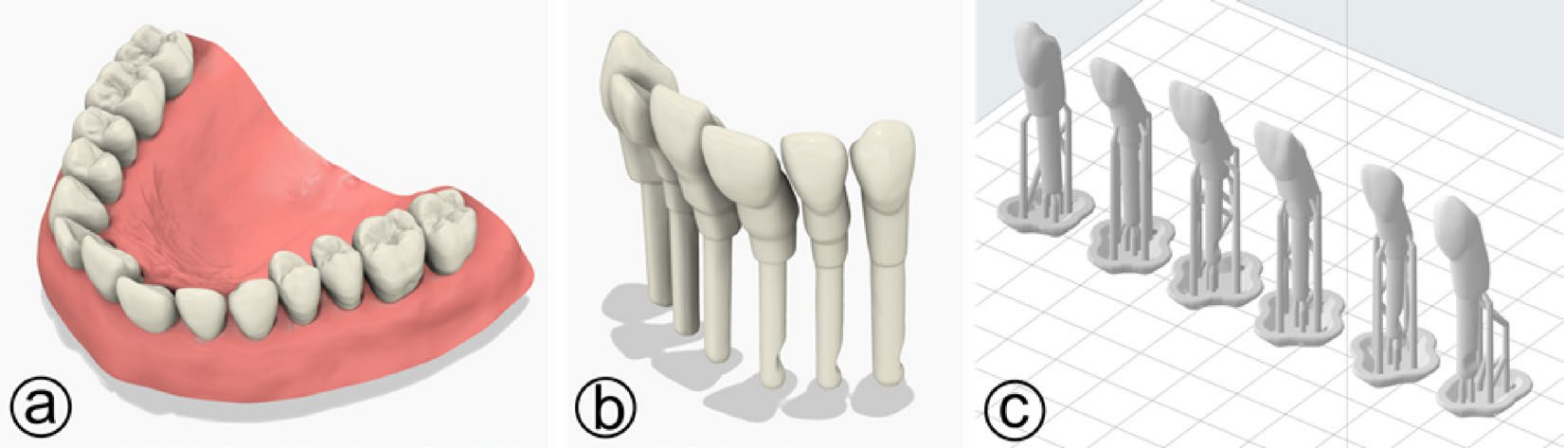
**(a)** 3D model before the repositioning of the anterior teeth; **(b)** finished construction of the printed teeth; **(c)** anterior teeth prepared for printing with included supports.

A printable set of maxillary anterior mobile teeth from the right to the left maxillary canine (FDI 13–23) were developed (Fig. 1a) and reproduce displacement and mobility from natural teeth. They were engineered with modified retention geometry (Fig. 1b) to allow controlled mobility and manual repositioning into an ideal arch, creating a realistic repositioning task before splint placement. The left and right maxillary canine were not modified to be stable for the splint like often seen in patients. Importantly, the teeth were designed to fit into a standard commercial study model (KaVo, Biberach an der Riß, Germany), enabling straightforward integration into existing phantom‑head infrastructure and facilitating reproduction at other institutions. For the production process the models were nested closely and printed on a Form 2 3D printer (Formlabs Inc., Somerville, MA, USA) (Fig. 1c). White photopolymer resin was used for printing (RS-F2-GPWH-04, Formlabs Inc.). After printing, the teeth were post-processed according to the manufacturer guidelines. This includes cleaning with isopropanol (Form Wash, Formlabs Inc.), UV curing (Form Cure, Formlabs Inc.) and manual removing of support structures.

### Training protocol

**Fig. 2:**
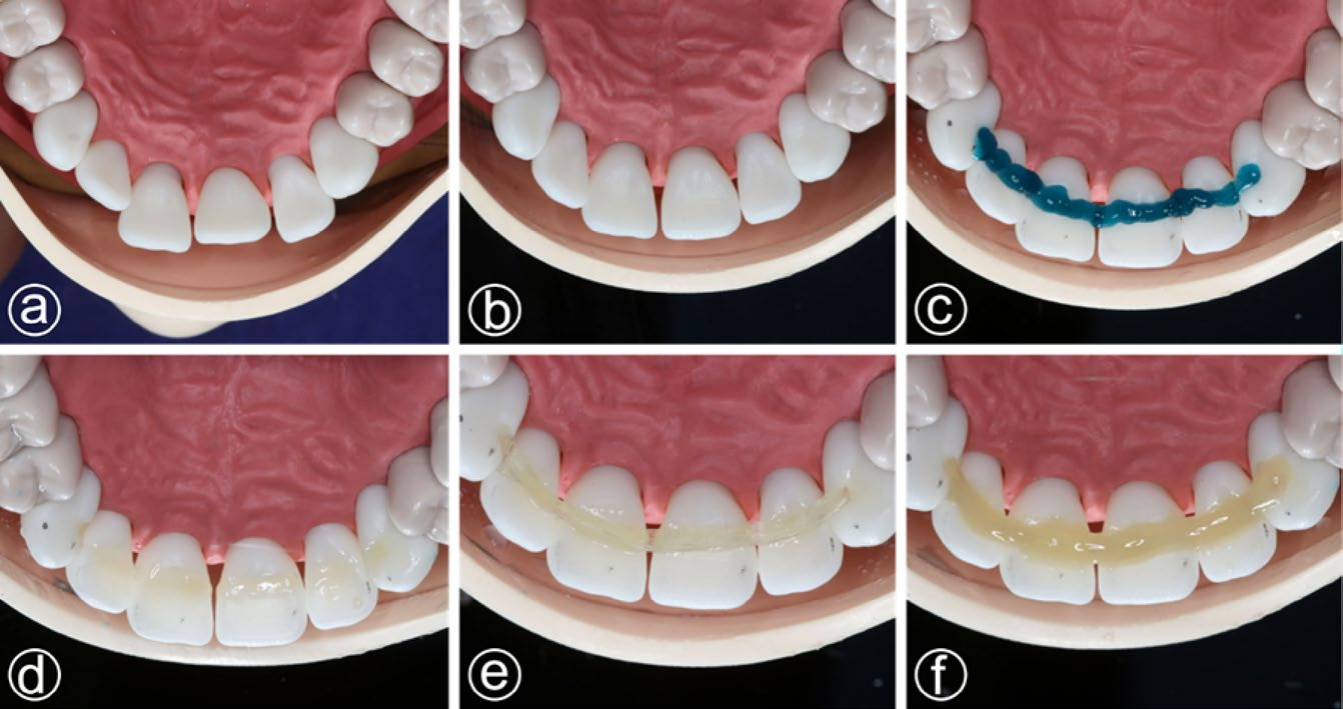
**(a)** printed teeth in the standard dental study model; **(b)** teeth after repositioning into the dental arch; **(c)** simulation of selective etching; **(d)** application of resin before the splint; **(e)** positioning of the splint into the resin; **(f)** completed splint after covering with resin.

The printed teeth were fitted into the standard dental study model (Fig. 2a). Then the teeth were manually repositioned to approximate the ideal arch and verified by testing the occlusion (Fig. 2b). A palatal channel with approximately 1.5 mm depth and width was prepared from the left to the right lateral incisor (FDI 12–22) and slightly extended to the canines. For realistic practice enamel etching (35% phosphoric acid, Ultradent, Brunnthal, Germany) (Fig. 2c) and adhesive were applied (OptiBond FL, Kerr, Orange, CA, USA), followed by a thin layer of flowable composite (Filtek Flowable Composite, 3 M ESPE, St. Paul, MN, USA) (Fig. 2d). One of the two splint materials was adapted to the channel length and embedded into it (Fig. 2e). After this the splint was fully covered with flowable composite, light-cured, finished and polished (Fig. 2f).

### Questionnaire

After the second course students completed an electronic questionnaire (EvaSys Electric Paper Evaluationssysteme GmbH, Lueneburg, Germany). The questionnaire was developed in collaboration with the Institute for Medical Teaching and Educational Research (IMLA) and had been used in prior evaluations of 3D‑printed dental training models. This includes questions about realism like tooth mobility and repositioning, material handling, learning effect and clinical relevance and included free-text questions about advantages, material preference and course organization (Table 1). Similar questionnaires have been used before by students to evaluate other studies with printed teeth^13^. The questionnaire was adapted to the present study. The evaluation was based on German school grading and therefore recorded on an anchored 6‑point ordinal scale (1 = Excellent, 2 = Good, 3 = Satisfactory, 4 = Adequate, 5 = Poor, 6 = Unsatisfactory). Because the scale is ordinal, results are reported as medians and interquartile ranges (IQR). The results of the distribution of grades were displayed in a bar chart and shown in percentage (Fig. 4). Free‑text responses were analyzed using descriptive content analysis. Three investigators independently reviewed responses, generated thematic categories, and reconciled discrepancies by consensus. Themes were counted and participants could contribute multiple themes per question. Initial inter-rater agreement prior to consensus was Fleiss’ κ = 0.83 interpreted as almost perfect agreement.

**Table 1:** The questionnaire for the evaluation of the course.

**1.**	**Personal data**
1.1.	Please enter your gender
1.2.	Please enter your age
**2.**	**Handling of the printed teeth**
2.1.	How realistic was the tooth loosening?
2.2.	How realistic was the repositioning into the dental arch?
**3.**	**Splinting of the printed teeth**
3.1.	Please evaluate the splinting exercise.
3.2.	Evaluate the handling of “everStick PERIO”.
3.3.	Evaluate the handling of “Ribbond Ultra”.
**4.**	**Evaluation of the learning process** Rate your preparation to perform a periodontal splint on a patient …
4.1.	… before the course.
4.2.	… after the course.
4.3.	Evaluate the learning effect of this course.
4.4.	Evaluate the practical relevance of this exercise.
4.5.	Do you prefer to use “everStick PERIO”.
4.6.	Do you prefer to use “Ribbond Ultra”.
**5.**	**Free text questions**
5.1.	What advantages do you see in this exercise?
5.2.	What could be improved in this excercise?
5.3.	Your opinion about the usage of “everStick PERIO”.
5.4.	Your opinion about the usage of “Ribbond Ultra”.

### 3D scanning and analysis

**Fig. 3:**
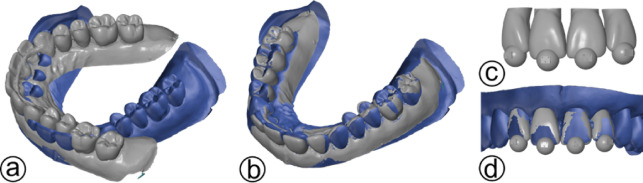
**(a)** Reference model (blue) and scanned student model (grey) before alignment. **(b)** Aligned models by initial coarse matching followed by local best‑fit on the posterior region. **(c)** Digitally added 6‑mm incisal spheres on the evaluated anterior teeth for distance‑based deviation measurements. **(d)** Example of matched reference teeth and scanned model with spheres.

A total of 86 splinted models and one instructor‑prepared reference model were scanned with an inEos X5 (Dentsply Sirona, Bensheim, Germany) dental laboratory scanner. STL meshes were generated and aligned with GOM Inspect (GOM, Braunschweig, Germany) (Fig. 3a). This was done by initial coarse alignment to the posterior teeth followed by local best-fit (Fig. 3b) to the CAD. To obtain reproducible landmark centers, 6 mm spheres were added digitally to the incisal edges of the evaluated anterior teeth. The centers of these spheres served as standardized points for distance‑based deviation measurements relative to the reference model (Fig. 3c). This approach provides robust, operator‑independent positional comparisons and reduces sensitivity to local mesh artefacts. The spheres also contained embedded semicircular recesses which served as reference features to support reproducible alignment and exploratory assessment of rotation (Fig. 3d). Limitations of the metric include reduced sensitivity to angular and rotational misalignment and the absence of a validated clinical threshold for acceptable deviation in this simulated setting.

Therefore, deviation values are interpreted as an educational performance presentation rather than a direct clinical endpoint and served as the primary quantitative outcome for statistical analysis.

### Statistical analysis

All data was analyzed using SPSS (Version 26; IBM Corp., Armonk, NY, USA). The primary outcome parameter for quantitative analysis was the positional deviation between incisal sphere centres of student‑prepared models and the instructor reference model, assessed after splinting. Because the cohort size was determined by class size, no a priori sample size calculation was performed. We therefore report a post hoc sensitivity analysis for the primary outcome to indicate the minimum within-participant effect size detectable with the available sample under α = 0.05. This analysis is provided for interpretability and does not convert non-significant findings into evidence of equivalence. Assuming a two‑sided α of 0.05 and a paired design with 43 participants, the study was sufficiently powered to detect a medium within‑participant effect size (Cohen’s dz ≈ 0.43) with 80% power. Smaller effects may therefore not have been detectable. The study is therefore framed as a pilot and exploratory evaluation. This is also common with other literature about this topic^1–7^. Several groups were not normally distributed, confirmed by Kolmogorov-Smirnov test. Because each participant completed both materials in a cross‑over design, primary comparisons were within‑participant. Accordingly, the Wilcoxon signed-rank test was used for paired non-parametric comparisons and for first versus second session comparisons. Where multiple teeth were evaluated, analyses were treated as exploratory and a Bonferroni‑adjusted alpha was applied to familywise comparisons.

## Results

### Questionnaire outcomes

**Fig. 4:**
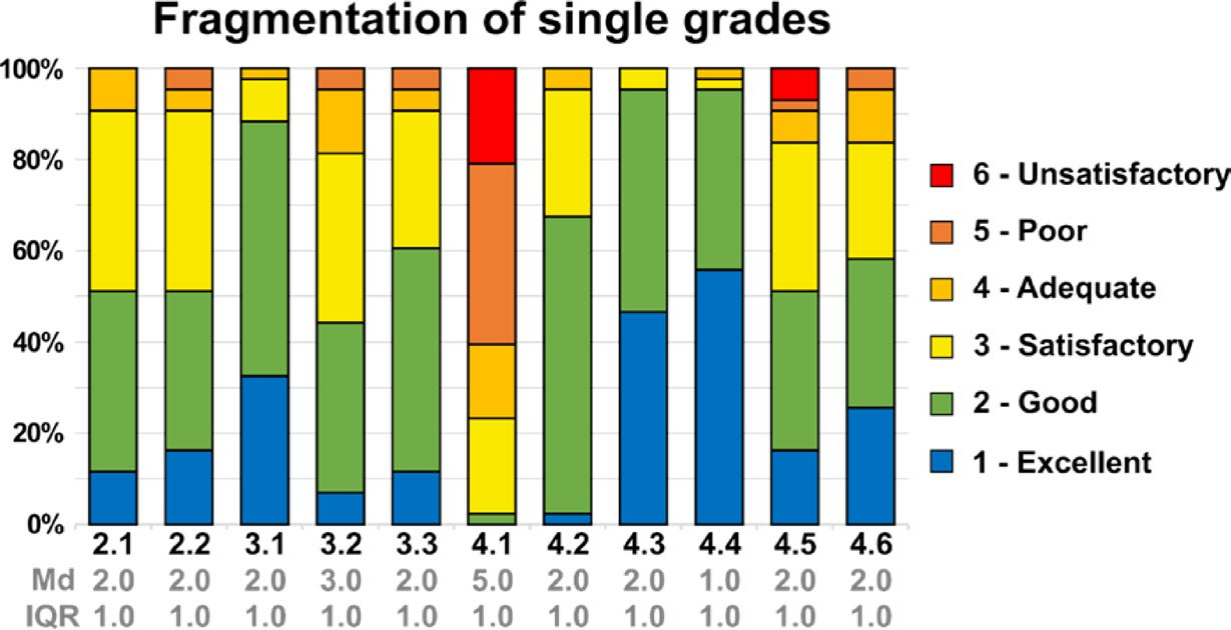
Results from the questionnaire in Table 1 as a stacked bar chart. The percentages of given grades are displayed. German school grading is shown at the right side. Median values (Md) and Interquartile Range (IQR) are displayed below corresponding questions.

The results are described descriptively, and important results are mentioned here. The Wilcoxon signed-rank test was applied where appropriate to compare responses. Figure 4 presents the stacked distribution bars. The realism of mobility and repositioning were both “good” (Fig. 4.2.1 and 4.2.2; Md 2.0, IQR 1.0). The course was rated “good” overall (Fig. 4.3.1; Md 2.0, IQR 1.0). No significant differences were observed for everStick PERIO and Ribbond Ultra in handling (Fig. 4.3.2 vs. 4.3.3 *p* = 0.23) or user preference (Fig. 4.4.5 vs. 4.4.6 *p* = 0.49). A substantial highly significant learning gain was reported by the students. Self-efficacy for doing a splint improved from “poor” (Fig. 4.4.1; Md 5.0, IQR 1.0) to a post‑course “good” (Fig. 4.4.2; Md 2.0, IQR 1.0; *p* < 0.001). Also, the clinical relevance was rated “excellent” (Fig. 4.4.4; Md 1.0, IQR 1.0). In the free-text was also no clear favorite for clinical usage, 23 preferred Ribbond Ultra and 18 everStick PERIO and 2 found them equal. For both materials the handling was criticized for different reasons but also positively evaluated by other students. For details see the following free-text analysis.

### Free‑text analysis

The results of the free-text questions were analyzed (Table 1.5.1-4.1). Similar answers were grouped and counted. The most frequently cited advantage was the realistic challenge of repositioning mobile teeth prior to splinting (*n* = 25), followed by the availability of script and video preparation materials (*n* = 12). Only minor suggestions for improvements were made, for example, refinements to realism (*n* = 3) and loosening of the teeth (*n* = 2).

### Advantages of the exercise

- realistic challenge of repositioning (*n* = 25).
- script and video material (*n* = 12).
- comparison of both materials (*n* = 6).
- good staff support (*n* = 6).
- using real material (*n* = 5).
- positive learning effect between sessions (*n* = 2).

Overall, two participants did not fill out question 5.1, resulting in a participation rate of 95.3%.

### Improvements for the exercise

- realism of the exercise (*n* = 3).
- no improvements needed (*n* = 2).
- degree of loosening is unrealistic (*n* = 2).
- occlusion in the phantom head not ideal (*n* = 2).
- more time for a discussion of the results (*n* = 2).

Overall, 15 participants did not fill out question 5.2, resulting in a participation rate of 65.1%.

### Opinions about everStick PERIO

- better handling and adaptation to the tooth (*n* = 18).
- difficult handling and adaptation into the prepared groove (*n* = 13).
- considered as too thick (*n* = 5).
- splinting was faster (*n* = 4) and easier (*n* = 2).
- too rigid/stiff (*n* = 3) or too sticky (*n* = 2).
- more stable and resistant (*n* = 3).

Overall, 6 participants did not fill out question 5.3, resulting in a participation rate of 86.0%.

### Opinions about ribbond ultra

- better handling, positioning and adaptation (*n* = 20).
- poorer handling, more difficult adaptation to the tooth (*n* = 13).
- material - and thus the splint - was thinner (*n* = 7).
- material was more flexible (*n* = 3).
- processing time was shorter or too short (*n* = 3).
- fiber-like consistency was criticized (*n* = 2).
- material was partially exposed during splint finishing (*n* = 2).
- better adhesion to the tooth or in the prepared groove (*n* = 2).

Overall, 5 participants did not fill out question 5.4, resulting in a participation rate of 88.4%.

### Geometric accuracy

**Fig. 5:**
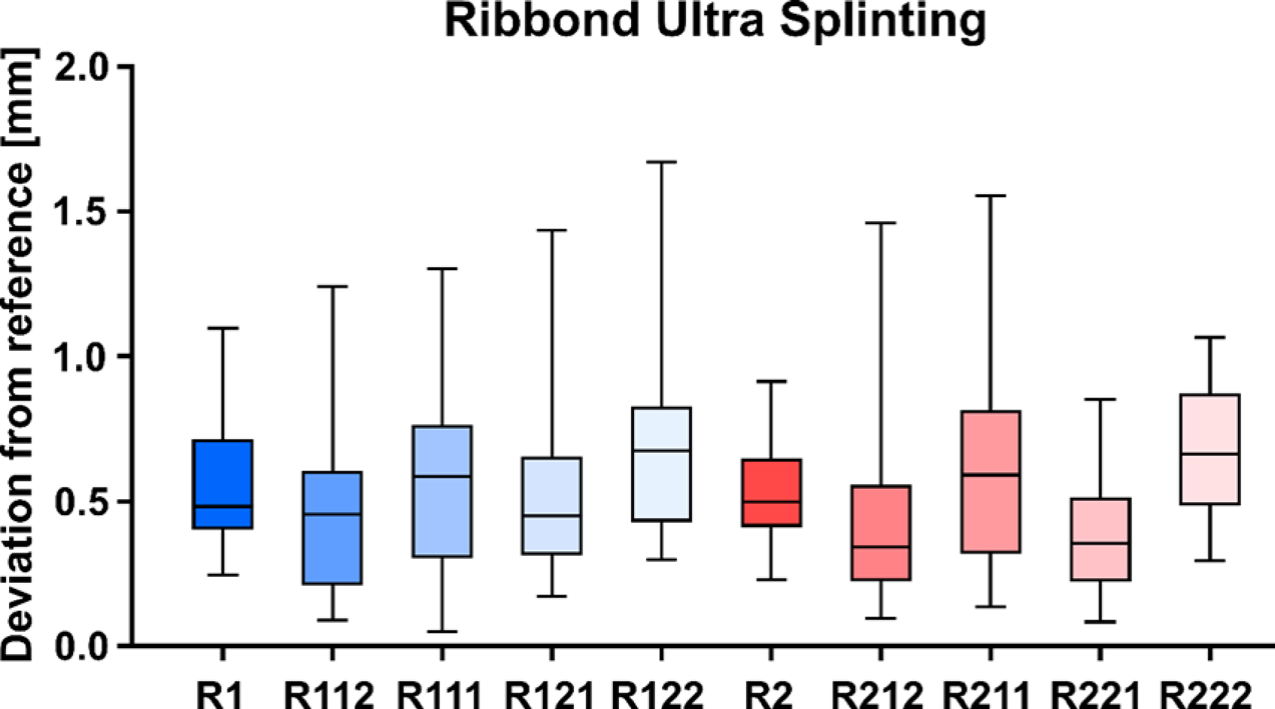
Boxplot illustrating deviations of grouped and individual teeth from the reference model after splinting with Ribbond Ultra. R denotes Ribbond Ultra; the first numeral indicates initial or secondary splinting. Subsequent numerals refer to the respective teeth according to the FDI tooth numbering system.

**Fig. 6:**
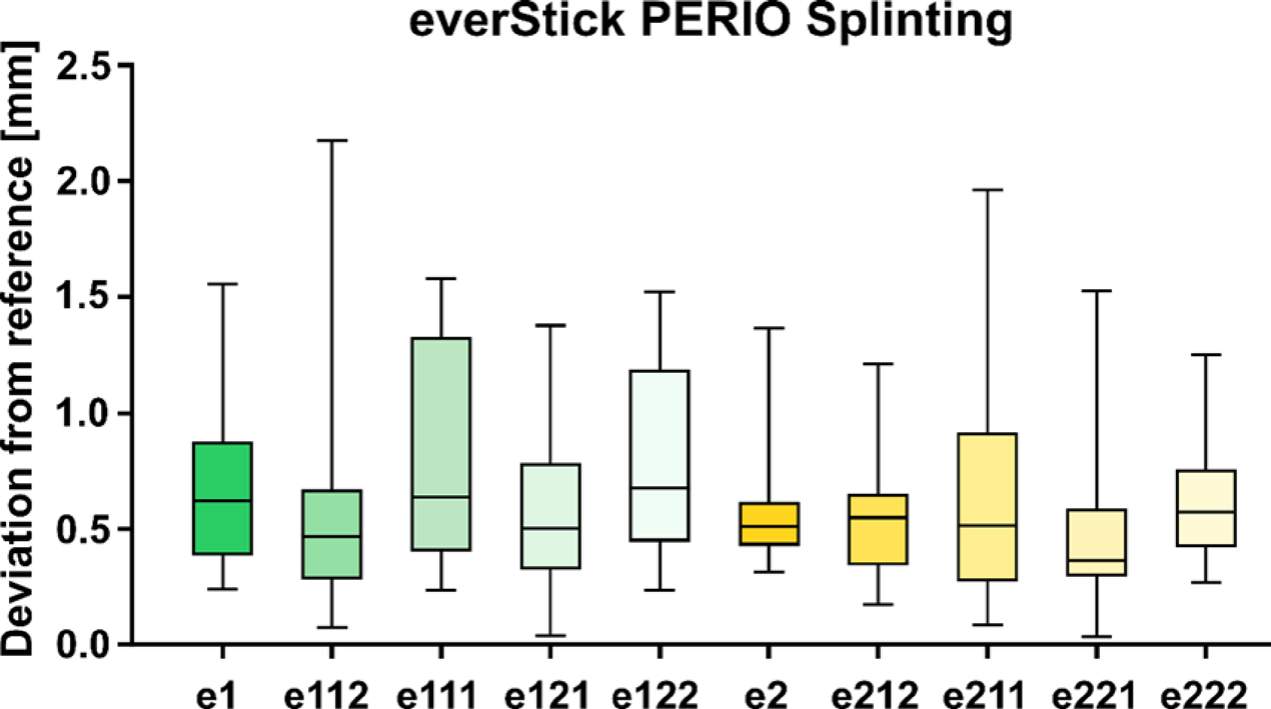
Boxplot illustrating deviations of grouped and individual teeth from the reference model after splinting with everStick PERIO. e denotes everStick PERIO; the first numeral indicates initial or secondary splinting. Subsequent numerals refer to the respective teeth according to the FDI tooth numbering system.

Statistical screening revealed that Kolmogorov-Smirnov test indicated that several distributions deviated from normality. Because of this non-parametric test was used. Median deviations between incisal sphere centers of student models and the reference model were calculated across teeth and sessions (Figs. 5 and 6). In this pilot cohort, we found no statistically detectable differences between materials or between the first and second session. These values should be interpreted as an objective performance indicator for arch conformity rather than a clinical acceptability threshold, which is not established for this simulation metric. However, two strong but non-significant differences with respect to tooth 21 was noticeable. When comparing different groups in relation to tooth 21, lower p-values were observed, which reached statistical significance prior to Bonferroni correction: R121 – R221 (*p* = 0.044) and e121 – R221 (*p* = 0.039). At the first attempt (R1) with Ribbond Ultra 0.48 mm deviation was measurable with a range from 0.25 to 1.10 mm and for the second (R2) 0.50 mm (0.24–0.92 mm) (Fig. 5). everStick PERIO had a deviation of 0.62 mm (0.24–1.56) for the first attempt (e1) and for the second attempt (e2) 0.51 mm (0.31–1.37) (Fig. 6).

## Discussion

This study introduces a 3D‑printed splinting model that specifically simulates anterior tooth mobility and the clinically relevant step of manual repositioning prior to fibre‑reinforced composite splint placement. The ability to repeatedly practice repositioning and stabilization in a standard phantom‑head environment distinguishes the model from fixed typodont approaches and supports scalable course delivery at low cost.

 Previously described 3D‑printed training models in dental education vary considerably in their design features and learning objectives. For example, Reymus et al.^5^ and Hoehne et al.^7,13^ describe interdisciplinary and modular models aimed at restorative and prosthodontic training, in which tooth position is fixed and the procedural focus lies on material handling and cavity or prosthetic preparation. Similarly, endodontic models by Kolling et al.^3^ and Marty et al.^6^ reproduce internal canal anatomy but do not incorporate tooth mobility or require repositioning prior to treatment. Surgical simulation models^2,4^ are typically based on patient-specific anatomy and aim at procedural rehearsal, again without modification of tooth position during the exercise.

In contrast to these approaches, the present model introduces controlled tooth mobility as a central design feature, requiring learners to actively reposition teeth to an ideal arch before splinting. This represents a distinct procedural step that is not addressed in the above models. Furthermore, while several previous models are institution-specific or designed as complete typodont replacements^1,2,4–7,14^, the present system is compatible with a standard commercial study model, enabling broader adoption without modification of existing infrastructure. Finally, unlike prior studies that rely primarily on subjective instructor or learner evaluation^1,13^, the present study incorporates an objective 3D deviation workflow to quantify repositioning accuracy.

Taken together, the novelty of the present model lies not in the use of 3D printing per se—which is well established—but in the combination of (i) reproducible tooth mobility, (ii) a required repositioning task prior to treatment, (iii) compatibility with standard models, and (iv) objective geometric performance assessment, none of which are jointly implemented in previously published training models to our knowledge.

In the questionnaire and geometric analysis were no material-dependent differences found. Because the cohort size was determined by class size and no a priori sample size calculation was performed, the study may be underpowered to detect small effects; therefore, non-significant findings should not be interpreted as proof of equivalence. On the other hand, the study represents a realistic number of participants comparable to normal course sizes. The post hoc sensitivity power analysis indicates that this pilot study was sufficiently powered to detect medium but not small differences in repositioning accuracy. Accordingly, non-significant findings should be interpreted as “no statistically detectable difference” rather than evidence of equivalence.

*For beginners*,* success is more influenced by fundamental procedural basics*,* such as tooth repositioning*,* moisture control*,* and composite coverage of the FRC system.* This is in common with reports that polyethylene ribbon and glass fibres can produce durable splints when correctly handled^8–12,15^. Under clinical conditions, long-term durability is affected by fibre architecture, impregnation quality, and composite coverage. Early performance is influenced mostly by user-dependent factors^16^. The students as novice beginners showed no accuracy differences between both FRC systems, but a small subjective handling gap was there. Ribbond Ultra was rated “good” and everStick PERIO “satisfactory”. This suggests that a small advantage is there for thinner and more adaptable ribbon in tight palatal channels. This was also mentioned in the free text questions by better handling, positioning and adaptation (*n* = 20). Students reported a clear confidence level after the training in splinting technique. This aligns with other simulation studies where enhanced confidence and reduced anxiety before patient treatment was reported^14,17–23^.

Our findings are congruent *with* competency‑based education principles. Structured practice and more challenging tasks support new skill acquisition. Self-assessment was promoted with the questionnaire, scanning and derived feedback^24,25^. The two training sessions were not enough to detect a significant learning effect. The consolidation phase of the learning curve was not completed. It can be assumed that additional sessions across weeks could enhance the consolidation and lead to measurable performance gains^26,27^. A variety of different difficulties can be generated in the future. Example for these are milder displacements, then a progression to greater rotation or elasticity.

Geometric accuracy was measured as distances between incisal-sphere centres of students against reference teeth. This method offers robustness against local mesh artefacts and variability in landmarking by operators. By this approach differences are collapsed into a single value, and angular misalignment is under-represented.

### Educational implications

The integration of this splinting exercise into clinical and preclinical curricula offers a structured opportunity to practice splinting with or without assistance, train with different materials and handling. The production cost of approximately 0.10€ including resin, consumables and work times are very low per tooth. The estimated per-tooth cost was calculated from the printed resin volume per tooth (0.79 ml; resin price €89/L; 0.07€ per tooth), plus consumables (2 L IPA 4€/L, around 4 batches usable; gloves 0.04€; 0.01€ per tooth), and post-processing labor (10 min/180 teeth at 15€/hour; 0.01€), for batch production with a batch size of 180 teeth per print run.

This makes it easy to perform repeated practice without financial pressure on students or institutions. Printing 31 six‑tooth sets at once in approximately 11 h 33 m makes it possible to produce course‑scale numbers on a single desktop printer (Form 2, Formlabs Inc.). Depending on the print technology with the fastest desktop printers this can be drastically reduced. For example, with a Form 4 3D printer (Formlabs Inc.) 28 six‑tooth sets can be printed in only 2 h. This new 3D-printed model is an effective training method before clinical patient treatment.

Future work should focus on expanding cohort sizes and incorporate more 3D metrics (e.g. angular deviations). Also, assisted versus unassisted workflows should be compared in regard to self-assessment and performance. While the here performed metrics have a focus on the arch conformity, clinical outcomes also rely on patient-specific factors like periodontal stability, occlusion, hygienical aspects and splint maintenance.

### Limitations

This study is a pilot educational evaluation conducted in a single cohort without a control group using conventional typodont training. Consequently, causal claims about superiority over established teaching methods cannot be made. Learner perception and self‑reported confidence are important but are subjective outcomes and may be influenced by volunteer and novelty effects. Only two training sessions were delivered, which may be insufficient to detect measurable performance improvement on objective metrics over time. The geometric deviation approach provides a reproducible positional control for tooth repositioning accuracy but underrepresents angular errors and does not directly capture clinical factors such as isolation, composite coverage quality, occlusion, or long‑term splint durability. Finally, the cohort size was determined by class size and may be underpowered to detect small between‑material differences; therefore, non‑significant findings should be interpreted as ‘no statistically detectable difference’ rather than proof of equivalence.

## Conclusion

This inexpensive and reproducible 3D‑printed mobile tooth model enables structured preclinical training of periodontal splinting under simulated mobility conditions. In this cohort, we found no statistically detectable differences between the two fibre‑reinforced composite systems on the selected questionnaire and positional deviation outcomes. Larger controlled studies with additional objective metrics are needed to determine comparative effectiveness and learning gains.

## Data Availability

The datasets generated and analyzed during the current study are available from the corresponding author on reasonable request.
